# Regional Differences in Women’s Basketball: A Comparison among Continental Championships

**DOI:** 10.3390/sports6030065

**Published:** 2018-07-20

**Authors:** Haruhiko Madarame

**Affiliations:** Department of Sports and Fitness, Shigakkan University, Nakoyama 55, Yokonemachi, Obu, Aichi 474-8651, Japan; madarame-tky@umin.ac.jp; Tel.: +81-562-46-1291

**Keywords:** basketball, game-related statistics, performance analysis, team sports

## Abstract

The aims of this study were (i) to compare basketball game-related statistics in women by region (Africa, America, Asia, Europe), and (ii) to identify characteristics that discriminate performances for each region. A total of 134 games from each continental championship held in 2017 were analyzed. A one-way ANOVA followed by a Bonferroni-adjusted pairwise comparison was performed to evaluate differences in each variable between the continents. A discriminant analysis was performed to identify game-related statistics that discriminate among the continents. The Asian and European championships overall showed similar performance profiles: Low numbers of possessions and turnovers, and high numbers of successful field goals and assists. However, the European championship was more closely contested than the Asian championship. The African championship was characterized by high numbers of possessions, free throws, and turnovers. The homogeneity of the American championship was low, and some of the cases have similarities with the African championship, whereas other cases have similarities with the European championship. On average, the American championship was characterized by low numbers of successful field goals and assists, and high numbers of steals and turnovers. It is suggested that women’s basketball games are played in a different manner in each region of the world.

## 1. Introduction

Basketball is one of the most popular sports in the world. As of 2018, the International Basketball Federation (FIBA) has 213 national member federations, and the FIBA estimates that there are 450 million players worldwide [1]. National teams compete in international competitions, such as the Olympic Basketball Tournament, the FIBA World Cup, and the FIBA Continental Cups. International competitions in basketball are governed by the FIBA, so that official games are played by the same rules with the same equipment anywhere in the world. However, regional differences in performance profiles have been reported in recent studies [2,3]. Ibáñez et al. [2] compared game-related statistics among continental championships for men held in 2015, and reported that each continent has a specific performance profile, which can be summarized as follows: Africa, high numbers of free throws, rebounds, steals, and fouls; America, a high number of field goal attempts; Asia, a high number of possessions and a low number of assists; and Europe, a low number of possessions and a high number of assists. These findings indicate that basketball games are played in a different manner in each continent.

From a practical perspective, the knowledge about regional differences in performance profiles would be useful for players and coaches of national teams preparing for international competitions. However, although international competitions are held not only for men, but also for women, previous studies on regional differences in basketball [2,3] have analyzed only men’s competitions. Sex differences in performance profiles have been reported in previous studies [4,5,6]. For example, Sampaio et al. [4] analyzed the world championships for both men and women held in 2002 and reported that men’s teams were discriminated from women’s teams by a higher percentage of blocks and a lower percentage of steals, suggesting that anthropometric differences between men and women might be attributable to the difference in performance profiles. Differences between men and women can also be found in the latest FIBA World Rankings [7,8]. In the men’s ranking, updated as of 28 February 2018 [7], the 10th-ranked Australia is the only country that ranks in the top 20 and belongs neither to America nor to Europe. In the women’s ranking, updated as of 27 August 2017 [8], however, the top 20 includes four countries from Asia (Australia, China, Japan, Korea) and two countries from Africa (Senegal, Angola) (Note that, although Australia belongs to FIBA Oceania, Oceanian championships have been merged with Asian championships since 2017, and Oceanian countries have been categorized into Asia in the FIBA World Rankings). Considering these facts, it is possible to assume that regional differences in performance profiles differ between men and women. If regional differences among continental championships for women are dissimilar to those for men, the previous findings on men [2,3] cannot be applied to women. Therefore, identifying regional differences in women’s basketball would be of help for players and coaches of women’s national teams to prepare for international competitions.

The number of studies on game-related statistics in women’s basketball has been increasing in recent years [9,10,11,12,13,14,15]. Game-related statistics have been analyzed to identify the relationship between performance indicators and match outcomes in international [9,10,11] and domestic [12,13,14] tournaments, and to identify performance indicators that discriminate starters from nonstarters in a professional league [15]. Although one study has investigated game-related statistics that discriminate winners from losers in both Asian and European women’s championships held in 2011, 2013, and 2015 [11], no studies have investigated regional differences in women’s basketball among four continental championships (Africa, America, Asia, Europe). Therefore, the aims of this study were (i) to compare basketball game-related statistics in women by region (Africa, America, Asia, Europe), and (ii) to identify characteristics that discriminate performances for each region.

## 2. Materials and Methods

Box scores of all 134 games in four continental championships for women held in 2017 (Table 1) were gathered from the official website of FIBA. 

Data reliability of the box scores was not assessed in this study. However, official box scores are treated as reliable in basketball studies [16,17], because the recording process is executed according to the regulations established by FIBA [18], and a high level of inter-rater reliability (kappa coefficient above 0.89) has been repeatedly confirmed [2,19,20,21]. Game-related statistics of each game were analyzed separately for the winning and losing teams, so that 268 cases were analyzed in total. The analyzed game-related statistics were as follows: 2- and 3-point field goals (successful and unsuccessful), free throws (successful and unsuccessful), defensive and offensive rebounds, assists, steals, turnovers, blocks, and fouls committed. Definitions of the statistics [18] are shown in Table 2.

To eliminate the effect of game rhythm, the variables were normalized to 100 game ball possessions [22]. Game ball possessions were calculated as an average of team ball possessions (TBP) of both teams [23]. TBP was calculated from field goal attempts (FGA), offensive rebounds (ORB), turnovers (TO), and free throw attempts (FTA) using the following equation [23]:

TBP = FGA − ORB + TO + 0.4 × FTA(1)

Statistical analyses were performed with R version 3.5.0 for Windows [24]. Statistical significance was set at *p* ≤ 0.05 unless otherwise stated. A one-way analysis of variance followed by a Bonferroni-adjusted pairwise comparison was performed to evaluate differences in each variable between the continents. Cohen’s *d* was calculated as an effect size and interpreted as follows: *d* = 0.20 to 0.49, small effect; *d* = 0.50 to 0.79, medium effect; *d* ≥ 0.80, large effect [25]. To identify game-related statistics that discriminate between the continents, a discriminant analysis was performed using R code ‘candis’ and ‘geneig’, which have been used in previous studies [3,6,11,26]. An absolute value of a structural coefficient (SC) greater than or equal to 0.30 was considered relevant for the discrimination between the continents [2,3].

## 3. Results

Significant *F*-values were obtained for point difference, team ball possessions, successful 2- and 3-point field goals, successful and unsuccessful free throws, assists, steals, turnovers, and fouls committed (Table 3). Large effect size differences between each continent were observed for point difference (Africa vs. Europe, *d* = 0.85; Asia vs. Europe, *d* = 0.97), team ball possessions (Africa vs. Europe, *d* =1.08; America vs. Europe, *d* = 1.45), unsuccessful free throws (Africa vs. Asia, *d* = 1.16; Africa vs. Europe, *d* = 0.82), assists (America vs. Asia, *d* = 1.03; America vs. Europe, *d* = 0.81), and fouls committed (America vs. Europe, *d* = 0.93; Asia vs. Europe, *d* = 1.05) (Table 3).

**Figure 1:**
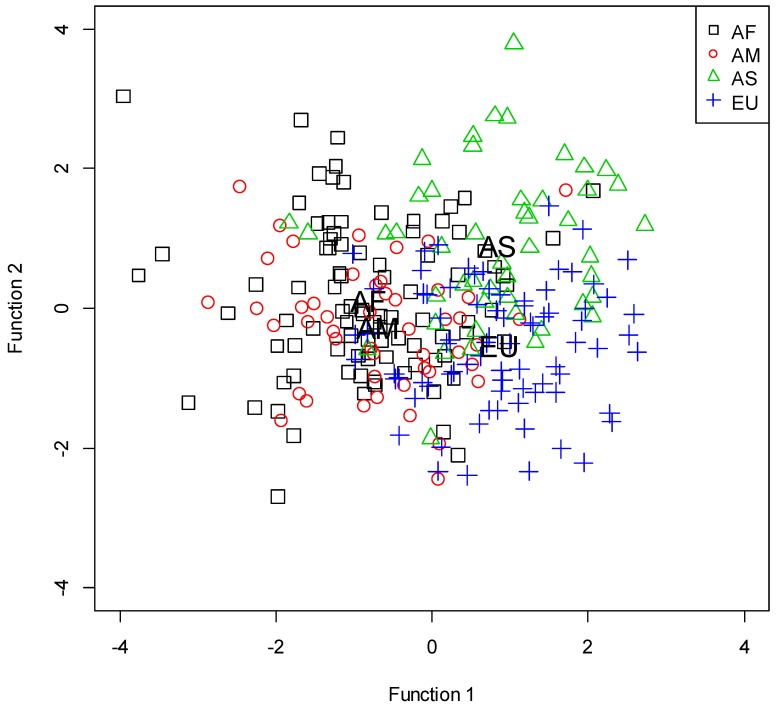
Territorial map of discriminant functions 1 and 2. AF, Africa; AM, America; AS, Asia; EU, Europe. Abbreviations plotted inside the figure indicate group centroids.

Classification results of the discriminant analysis are presented in Table 4. The total correct classification rate was 63.1%. Three significant functions were obtained from the discriminant analysis (Table 5). The territorial map of discriminant functions 1 and 2 is shown in Figure 1. The African and American championships were discriminated from the Asian and European championships by team ball possessions, unsuccessful free throws, assists, and turnovers (Function 1). The Asian championship was discriminated from the European championship by team ball possessions, assists, and fouls committed (Function 2). The African championship was discriminated from the American championship by successful free throws, unsuccessful free throws, and fouls committed (Function 3).

## 4. Discussion

This study analyzed game-related statistics of four continental championships for women held in 2017. The results showed that (a) significant differences among the continents were observed by ANOVA for 10 of 16 variables; (b) large effect size differences were observed for point difference, team ball possessions, unsuccessful free throws, assists, and fouls committed; (c) three significant functions that discriminate among the continents were obtained from the discriminant analysis. These results indicate that each continental championship has a specific performance profile and suggests that women’s basketball games are played in a different manner in each region of the world.

The discriminant analysis showed that the correct classification rate for the European championship was the highest among the four continental championships. This result indicates a high homogeneity of the European championship. The mean point difference between the winning and losing teams in the European championship was the smallest among the four continental championships. In the latest FIBA World Ranking [8], a total of nine European countries, which is the highest number among the four continents, are listed in the top 20. It should be evident that the European championship was the most closely contested championship. One of the performance profiles of the European championship can be seen in ball possessions, which showed the lowest number among the four continental championships. A low number of possessions indicates that the game pace was slow, suggesting that European teams tended to run a set offense. This assumption is also supported by the fact that the number of assists, which has been considered as an indicator of a well-organized offense [16,27,28], in the European championship, was relatively high among the four continental championships (second to the Asian championship). These performance profiles were consistent with previously reported findings in continental championships for senior [2] and junior [3] men. It is suggested that the basic performance profiles of European basketball are common to both sexes.

In contrast to the European championship, the mean point difference between the winning and losing teams and the number of ball possessions in the African championship were the largest among the four continental championships. Among the 12 teams that took part in the African championship, the highest ranked team in the latest FIBA World Ranking was the 17th-ranked Senegal, whereas the lowest was the 75th-ranked Central African Republic [8]. This huge disparity among the participating teams was likely a cause of the large point difference between winning and losing teams in the African championship. Although a high number of possessions should result in a high number of offensive opportunities, the numbers of successful 2- and 3-point field goals in the African championship were relatively low among the four continental championships (the second lowest and the lowest, respectively). However, the numbers of free throws and turnovers in the African championship were relatively high among the four continental championships (the highest and the second highest, respectively). High numbers of free throws [2,3] and turnovers [2] in African games have also been reported in previous studies on men’s championships. It is likely that players tended to lose possession before attempting a field goal or to be fouled during a shot in the African championship.

The correct classification rate for the Asian championship was relatively low among the four continental championships (52.1%, the second lowest), and 33.3% of the cases were misclassified into the European championship. This result indicates that a considerable number of the cases in the Asian championship have similar characteristics to the European championship. High numbers of successful field goals and assists, and low numbers of possessions, free throws and turnovers were common to the Asian and European championships. These findings were interesting because, unlike the European and African championships, the performance profiles of the Asian championship for women were largely different from Asian championships for senior [2] and junior [3] men. It has been suggested, because of a high number of possessions and a low number of assists, that the game pace is fast, and many points are scored after individual actions in Asian championships for men [2,3]. However, the present study showed that the Asian championship for women was characterized by a slow pace and well-organized offense. The difference in performance profiles between men and women may be related to differences in competitive performances in international competitions. As noted in the Introduction, four Asian countries are listed in the top 20 of the latest FIBA World Ranking for women [8], whereas only one country is listed in the top 20 of the FIBA World Ranking for men [7]. In addition, Asian women have shown better performances than Asian men in the Olympic Basketball Tournaments [29,30] and the FIBA World Cups [31,32].

The correct classification rate for the American championship was the lowest (43.8%) among the four continental championships. In the American championship, 29.2% of the cases were misclassified into the African championship, and 22.9% of the cases were misclassified into the European championship. These results indicate that the homogeneity of the American championship was low, and some of the cases have similarities with the African championship, whereas other cases have similarities with the European championship. Although it is difficult to clarify the performance profiles of the American championship due to the low homogeneity, some characteristics specific to the American championship could be found in game-related statistics. The numbers of points scored, successful 2-point field goals, and assists in the American championship were the lowest among the four continental championships, whereas the numbers of steals and turnovers in the American championship were the highest among the four continental championships. These findings were inconsistent with previously reported findings in continental championships for men held in 2015 [2], in which the numbers of points scored and successful 2-point field goals in the American championship were the highest among the four continental championships. In contrast to the American championship for men, the American championship for women seems to be a defense-oriented, low-scoring championship.

Although this study provides novel information that each continental championship has a specific performance profile, it is not without limitations. Since the methodology is purely quantitative in nature, qualitative elements of the game, such as types of offense [33,34] and defense [35,36], remain unrevealed. Future studies on qualitative elements of the game would compensate for this limitation and provide further understanding of regional differences in women’s basketball.

From a practical perspective, this study will help players and coaches of women’s national teams prepare for international competitions. At international competitions, national teams are required to play games against relatively unfamiliar teams in a short period of time. Detailed information about opponent teams can only be obtained through specific scouting of each opponent. However, basic information about opponent teams can be obtained from this study based on the region of the world where each opponent belongs.

## 5. Conclusions

This study identified regional differences in basketball games among four continental championships for women held in 2017. The Asian and European championships overall showed similar performance profiles: Low numbers of possessions and turnovers, and high numbers of successful field goals and assists. However, the European championship was more closely contested than the Asian championship. The African championship was characterized by high numbers of possessions, free throws, and turnovers, and a low number of successful field goals. The homogeneity of the American championship was low, and some of the cases have similarities with the African championship, whereas other cases have similarities with the European championship. On average, the American championship was characterized by low numbers of successful field goals and assists, and high numbers of steals and turnovers. It is suggested that women’s basketball games are played in a different manner in each region of the world.

## Figures and Tables

**Table 1:** Sample characteristics.

Region	Teams	Games	Cases
Africa	12 (Angola, Central African Republic, Cameroon, Cote d’Ivoire, Dem. Rep. of Congo, Egypt, Guinea, Mali, Mozambique, Nigeria, Senegal, Tunisia)	46	92
America	10 (Argentina, Brazil, Canada, Colombia, Cuba, Mexico, Paraguay, Puerto Rico, Venezuela, Virgin Islands)	24	48
Asia	8 (Australia, China, Chinese Taipei, D.P.R. of Korea, Japan, Korea, New Zealand, Philippines)	24	48
Europe	16 (Belarus, Belgium, Czech Republic, France, Greece, Hungary, Italy, Latvia, Montenegro, Russia, Serbia, Slovak Republic, Slovenia, Spain, Turkey, Ukraine)	40	80
Total	46	134	268

**Table 2:** Definitions of game-related statistics according to International Basketball Federation (FIBA) statisticians’ manual.

Statistics	Definitions
2-point field goals	A 2-point field goal attempt is charged to a player any time he shoots, throws, or tips a live ball at his opponent’s basket in an attempt to score a goal from the 2-point field goal area.
3-point field goals	A 3-point field goal attempt is charged to a player any time he shoots, throws, or tips a live ball at his opponent’s basket in an attempt to score a goal from the 3-point field goal area.
Free throws	A free throw is an opportunity given to a player to score one point, uncontested, from a position behind the free-throw line and inside the semi-circle.
Rebounds	A rebound is the controlled recovery of a live ball by a player or a team being entitled to the ball for a throw-in after a missed field goal attempt or last free throw attempt.
Assists	An assist is a pass that leads directly to a team-mate scoring. Scoring includes free throws. If the player who receives the pass is fouled in the act of shooting and makes at least one free throw, an assist is awarded in the same way as for a field goal made.
Steals	A steal is charged to a defensive player when his action causes a turnover by an opponent. A steal must always include touching the ball, but does not necessarily have to be controlled.
Turnovers	A turnover is a mistake by an offensive player or team that results in the defensive team gaining possession of the ball.
Blocks	A blocked shot is charged to a player any time he appreciably makes contact with the ball to alter the flight of a field goal attempt and the shot is missed.
Fouls committed	A foul is an infraction of the rules concerning illegal personal contact with an opponent and/or unsportsmanlike behavior.

**Table 3:** Game-related statistics of each continental championship with results of the ANOVA and post hoc comparisons.

Statistics	AF		AM		AS		EU		ANOVA		AF-AM		AF-AS		AF-EU		AM-AS		AM-EU		AS-EU	
	Mean	SD	Mean	SD	Mean	SD	Mean	SD	*F*	*P*	*P*	*d*	*P*	*d*	*P*	*d*	*P*	*d*	*P*	*d*	*P*	*d*
PTS	66.5	19.2	63.6	12.2	70.2	17.8	65.1	10.4	1.66	0.18	1.00	0.17	1.00	0.20	1.00	0.09	0.23	0.43	1.00	0.13	0.43	0.38
PD	25.3	19.4	16.0	11.9	24.8	17.9	12.3	8.4	13.07	**<0.01**	**<0.01**	0.54	1.00	0.03	**<0.01**	**0.85**	**0.03**	0.57	1.00	0.38	**<0.01**	**0.97**
TBP	78.2	7.7	78.1	5.4	75.1	6.3	71.5	3.9	20.48	**<0.01**	1.00	0.02	**0.03**	0.43	**<0.01**	**1.08**	0.11	0.50	**<0.01**	**1.45**	**<0.01**	0.73
S2P	23.9	8.2	22.9	6.2	28.0	9.3	26.3	5.8	5.32	**<0.01**	1.00	0.14	**0.01**	0.48	0.23	0.33	**<0.01**	0.65	0.07	0.57	1.00	0.23
U2P	35.2	7.7	34.8	9.6	34.6	8.6	35.3	7.9	0.09	0.96	1.00	0.05	1.00	0.07	1.00	0.01	1.00	0.02	1.00	0.06	1.00	0.08
S3P	6.2	3.7	6.7	4.0	8.0	4.1	7.5	3.4	3.14	**0.03**	1.00	0.12	**0.05**	0.46	0.13	0.38	0.53	0.32	1.00	0.24	1.00	0.12
U3P	16.8	6.6	18.0	5.6	17.5	7.4	17.2	5.6	0.42	0.74	1.00	0.19	1.00	0.11	1.00	0.07	1.00	0.07	1.00	0.14	1.00	0.04
SFT	18.5	7.2	15.8	5.4	13.7	5.9	15.9	6.8	6.06	**<0.01**	0.15	0.40	**<0.01**	0.70	0.07	0.36	0.67	0.38	1.00	0.02	0.38	0.35
UFT	10.1	4.8	6.8	3.1	5.1	3.1	6.5	3.9	21.09	**<0.01**	**<0.01**	0.78	**<0.01**	**1.16**	**<0.01**	**0.82**	0.26	0.54	1.00	0.08	0.37	0.38
DRB	36.7	7.6	39.3	8.3	36.4	8.5	37.9	5.9	1.68	0.17	0.32	0.33	1.00	0.04	1.00	0.18	0.37	0.34	1.00	0.20	1.00	0.22
ORB	17.8	7.5	15.8	6.5	15.9	7.3	16.2	7.1	1.20	0.31	0.78	0.27	0.94	0.24	0.98	0.21	1.00	0.02	1.00	0.06	1.00	0.04
AST	18.9	7.9	16.8	6.0	25.3	10.0	21.6	5.8	12.42	**<0.01**	0.69	0.29	**<0.01**	0.74	0.13	0.38	**<0.01**	**1.03**	**<0.01**	**0.81**	**0.04**	0.49
STL	11.7	5.1	12.0	5.2	9.6	5.0	10.8	4.3	2.65	**0.05**	1.00	0.06	0.10	0.42	1.00	0.19	0.10	0.47	1.00	0.26	1.00	0.26
TO	24.2	7.2	24.5	6.2	20.3	4.8	20.9	5.2	7.88	**<0.01**	1.00	0.04	**<0.01**	0.60	**<0.01**	0.51	**<0.01**	0.75	**<0.01**	0.63	1.00	0.12
BLK	2.9	2.7	3.2	2.5	3.5	2.5	3.6	2.6	1.19	0.31	1.00	0.10	1.00	0.23	0.50	0.26	1.00	0.13	1.00	0.16	1.00	0.03
FC	24.2	6.2	23.3	5.4	22.7	5.1	28.3	5.4	13.81	**<0.01**	1.00	0.16	0.72	0.27	**<0.01**	0.69	1.00	0.11	**<0.01**	**0.93**	**<0.01**	**1.05**

PTS, points scored; PD, point difference; TBP, team ball possessions; S2P, successful 2-point field goals; U2P, unsuccessful 2-point field goals; S3P, successful 3-point field goals; U3P, unsuccessful 3-point field goals; SFT, successful free throws; UFT, unsuccessful free throws; DRB, defensive rebounds; ORB, offensive rebounds; AST, assists; STL, steals; TO, turnovers; BLK, blocks; FC, fouls committed. *p* ≤ 0.05 and *d* ≥ 0.80 are shown in bold.

**Table 4:** Classification results of discriminant analysis.

Calculation	Region	Predicted				Total
		AF	AM	AS	EU	
Count	AF	**62**	10	8	12	92
	AM	14	**21**	2	11	48
	AS	6	1	**25**	16	48
	EU	10	3	6	**61**	80
Percentage	AF	**67.4**	10.9	8.7	13.0	100
	AM	29.2	**43.8**	4.2	22.9	100
	AS	12.5	2.1	**52.1**	33.3	100
	EU	12.5	3.8	7.5	**76.3**	100

AF, Africa; AM, America; AS, Asia; EU, Europe. Correct classifications are shown in bold.

**Table 5:** Discriminant functions with structural coefficients (SC) for each variable.

Statistics	Function 1	Function 2	Function 3
Eigenvalue	0.68	0.25	0.15
Wilks’ Lambda	0.42	0.70	0.87
Chi-square	226.1	93.0	35.9
Proportion of trace (%)	63.0	23.1	13.9
Canonical correlation	0.63	0.45	0.36
*p*	<0.01	<0.01	<0.01
Team ball possessions	**−0.54**	**0.37**	0.16
Successful 2-point field goals	0.27	0.18	−0.09
Unsuccessful 2-point field goals	0.00	−0.04	−0.06
Successful 3-point field goals	0.22	0.06	0.12
Unsuccessful 3-point field goals	0.02	0.00	0.18
Successful free throws	−0.24	−0.16	**−0.41**
Unsuccessful free throws	**−0.47**	−0.09	**−0.76**
Defensive rebounds	−0.01	−0.20	0.25
Offensive rebounds	−0.09	0.02	−0.24
Assists	**0.38**	**0.39**	−0.16
Steals	−0.18	−0.18	0.01
Turnovers	**−0.36**	−0.09	0.05
Blocks	0.14	−0.04	0.07
Fouls committed	0.25	**−0.61**	**−0.40**

|SC| ≥ 0.30 was considered relevant for discrimination (shown in bold).

## References

[1] International Basketball Federation (FIBA) Presentation, Facts & Figures. http://www.fiba.basketball/presentation#tab=element_2_1.

[2] Ibáñez S.J., González-Espinosa S., Feu S., García-Rubio J. (2018). Basketball without borders? Similarities and differences among Continental Basketball Championships. RICYDE. Rev. Int. Cienc. Deporte.

[3] Madarame H. (2018). Are regional differences in basketball already established in under-18 games?. Motriz Rev. Educ. Fis..

[4] Sampaio J., Godoy S.I., Feu S. (2004). Discriminative power of basketball game-related statistics by level of competition and sex. Percept. Mot. Skills.

[5] Gómez M.A., Lorenzo A., Ibáñez S.J., Sampaio J. (2013). Ball possession effectiveness in men’s and women’s elite basketball according to situational variables in different game periods. J. Sports Sci..

[6] Madarame H. (2018). Age and sex differences in game-related statistics which discriminate winners from losers in elite basketball games. Motriz Rev. Educ. Fis..

[7] International Basketball Federation (FIBA) FIBA World Ranking presented by Nike, Men. http://www.fiba.com/rankingmen.

[8] International Basketball Federation (FIBA) FIBA World Ranking presented by Nike, Women. http://www.fiba.basketball/rankingwomen.

[9] Conte D., Lukonaitiene I. (2018). Scoring strategies differentiating between winning and losing teams during FIBA EuroBasket Women 2017. Sports (Basel).

[10] Leicht A., Gomez M., Woods C. (2017). Team performance indicators explain outcome during women’s basketball matches at the Olympic Games. Sports (Basel).

[11] Madarame H. (2018). Defensive rebounds discriminate winners from losers in European but not in Asian women’s basketball championships. Asian J. Sports Med..

[12] Gómez M.A., Lorenzo A., Sampaio J., Ibáñez S.J. (2006). Differences in game-related statistics between winning and losing teams in women’s basketball. J. Hum. Mov. Stud..

[13] Moreno E., Gómez M.A., Lago C., Sampaio J. (2013). Effects of starting quarter score, game location, and quality of opposition in quarter score in elite women’s basketball. Kinesiology.

[14] Şentuna M., Şentuna N., Özdemir N., Serter K., Özen G. (2018). The investigation of the effects of some variables in the playoff games played in Turkey Women’s Basketball Super League between 2013–2017 on winning and losing. Phys. Educ. Stud..

[15] Gómez M.A., Lorenzo A., Ortega E., Sampaio J., Ibáñez S.J. (2009). Game related statistics discriminating between starters and nonstarters players in Women’s National Basketball Association League (WNBA). J. Sports Sci. Med..

[16] Garcia J., Ibáñez S.J., De Santos R.M., Leite N., Sampaio J. (2013). Identifying basketball performance indicators in regular season and playoff games. J. Hum. Kinet..

[17] Paulauskas P., Masiulis N., Vaquera A., Figueira B., Sampaio J. (2018). Basketball game-related statistics that discriminate between European players competing in the NBA and in the Euroleague. J. Hum. Kinet..

[18] International Basketball Federation (2016). FIBA Statisticians’ Manual 2016.

[19] Sampaio J., Lago C., Drinkwater E.J. (2010). Explanations for the United States of America’s dominance in basketball at the Beijing Olympic Games (2008). J. Sports Sci..

[20] Ibáñez S.J., García-Rubio J., Gómez M.A., Gonzalez-Espinosa S. (2018). The impact of rule modifications on elite basketball teams’ performance. J. Hum. Kinet..

[21] Gómez M.A., Avugos S., Ángel Oñoro M., Lorenzo Calvo A., Bar-Eli M. (2018). Shaq is not alone: Free-throws in the final moments of a basketball game. J. Hum. Kinet..

[22] Sampaio J., Janeira M. (2003). Statistical analyses of basketball team performance: understanding teams’ wins and losses according to a different index of ball possessions. Int. J. Perform. Anal. Sport.

[23] Oliver D. (2004). Watching a game: Offensive score sheets. Basketball on Paper: Rules and Tools for Performance Analysis.

[24] R Core Team (2018). R: A Language and Environment for Statistical Computing.

[25] Cohen J. (1992). A power primer. Psychol. Bull..

[26] Madarame H. (2017). Game-related statistics which discriminate between winning and losing teams in Asian and European men’s basketball championships. Asian J. Sports Med..

[27] Lorenzo A., Gómez M.A., Ortega E., Ibáñez S.J., Sampaio J. (2010). Game related statistics which discriminate between winning and losing under-16 male basketball games. J. Sports Sci. Med..

[28] Gómez M.A., Ibáñez S.J., Parejo I., Furley P. (2017). The use of classification and regression tree when classifying winning and losing basketball teams. Kinesiology.

[29] International Olympic Committee (IOC) Basketball Women. https://www.olympic.org/basketball/basketball-women.

[30] International Olympic Committee (IOC) Basketball Men. https://www.olympic.org/basketball/basketball-men.

[31] International Basketball Federation (FIBA) All Time Medalists: Results of the 17 editions of “FIBA World Championship for Women”. http://www.fiba.com/world/women/2014/alltimemedalists.

[32] International Basketball Federation (FIBA) All Time Medalists: Results of the 18 editions of “FIBA Basketball World Cup”. http://www.fiba.com/basketballworldcup/2019/alltimemedalists.

[33] Ciampolini V., Ibáñez S.J., Nunes E.L.G., Borgatto A.F., Nascimento J.V.D. (2017). Factors associated with basketball field goals made in the 2014 NBA finals. Motriz Rev. Educ. Fis..

[34] Conte D., Favero T.G., Niederhausen M., Capranica L., Tessitore A. (2017). Determinants of the effectiveness of fast break actions in elite and sub-elite Italian men’s basketball games. Biol. Sport.

[35] Gómez M.A., Lorenzo A., Ibáñez S.J., Ortega E., Leite N., Sampaio J. (2010). An analysis of defensive strategies used by home and away basketball teams. Percept. Mot. Skills.

[36] Gómez M.A., Tsamourtzis E., Lorenzo A. (2006). Defensive systems in basketball ball possessions. Int. J. Perform. Anal. Sport.

